# Evidence of brain target engagement in Parkinson’s disease and multiple sclerosis by the investigational nanomedicine, CNM-Au8, in the REPAIR phase 2 clinical trials

**DOI:** 10.1186/s12951-023-02236-z

**Published:** 2023-12-13

**Authors:** Jimin Ren, Richard B. Dewey, Austin Rynders, Jacob Evan, Jeremy Evan, Shelia Ligozio, Karen S. Ho, Peter V. Sguigna, Robert Glanzman, Michael T. Hotchkin, Richard B. Dewey, Benjamin M. Greenberg

**Affiliations:** 1 University of Texas Southwestern Medical Center, Department of Neurology, 5323 Harry Hines Blvd, Dallas, TX 75390 USA; 2 Present Address: Parkinson’s Disease and Movement Disorders Center, Boca Raton, FL 33486 USA; 3 Clene Nanomedicine, Inc., 6550 S Millrock Dr., Suite G50, Salt Lake City, UT 84121 USA; 4 Instat Clinical Research, A Veristat Company, 1 Wilson St., Chatham, NJ 07928 USA

**Keywords:** Catalytic gold nanotherapeutic, Target engagement, Drug development, Parkinson’s Disease, Multiple sclerosis, Neuroimaging, Brain energy metabolites, Magnetic resonance spectroscopy

## Abstract

**Background:**

Impaired brain energy metabolism has been observed in many neurodegenerative diseases, including Parkinson’s disease (PD) and multiple sclerosis (MS). In both diseases, mitochondrial dysfunction and energetic impairment can lead to neuronal dysfunction and death. CNM-Au8® is a suspension of faceted, clean-surfaced gold nanocrystals that catalytically improves energetic metabolism in CNS cells, supporting neuroprotection and remyelination as demonstrated in multiple independent preclinical models. The objective of the Phase 2 REPAIR-MS and REPAIR-PD clinical trials was to investigate the effects of CNM-Au8, administered orally once daily for twelve or more weeks, on brain phosphorous-containing energy metabolite levels in participants with diagnoses of relapsing MS or idiopathic PD, respectively.

**Results:**

Brain metabolites were measured using 7-Tesla ^31^P-MRS in two disease cohorts, 11 participants with stable relapsing MS and 13 participants with PD (n = 24 evaluable post-baseline scans). Compared to pre-treatment baseline, the mean NAD^+^/NADH ratio in the brain, a measure of energetic capacity, was significantly increased by 10.4% after 12 + weeks of treatment with CNM-Au8 (0.584 units, SD: 1.3; *p* = 0.037, paired t-test) in prespecified analyses of the combined treatment cohorts. Each disease cohort concordantly demonstrated increases in the NAD^+^/NADH ratio but did not reach significance individually (*p* = 0.11 and *p* = 0.14, PD and MS cohorts, respectively). Significant treatment effects were also observed for secondary and exploratory imaging outcomes, including β-ATP and phosphorylation potential across both cohorts.

**Conclusions:**

Our results demonstrate brain target engagement of CNM-Au8 as a direct modulator of brain energy metabolism, and support the further investigation of CNM-Au8 as a potential disease modifying drug for PD and MS.

**Supplementary Information:**

The online version contains supplementary material available at 10.1186/s12951-023-02236-z.

## Background

Brain function is critically dependent on a continuous supply of chemical energy in the form of adenosine triphosphate (ATP). Advanced age, a known risk factor for most neurodegenerative diseases, is associated with compromised energy metabolism, such as decreased glucose uptake and decreased mitochondrial electron transport chain activity, leading to lower ATP availability in the brain. These metabolic impairments are compounded in neurodegenerative disease, and are often detectable in advance of clinical symptom onset [1–3]. In neurodegenerative disease, a vicious cycle occurs in which energetic deficits exacerbate oxidative, proteostatic, and neuroinflammatory stressors, which in turn lead to further energetic impairment and neurodegeneration [1, 4].

Measurement of brain energy metabolites, in particular nicotinamide adenine dinucleotide (oxidized form: NAD^+^, reduced form: NADH), and ATP, have revealed important insights into the energetic deficits in neurodegenerative disease. Not only do NAD^+^ and NADH comprise the essential redox couple for major cellular ATP-generating reactions, namely oxidative phosphorylation and glycolysis, but they also serve as key intracellular energy sensors that coordinate nutrient availability and cellular energy status with ATP production [5]. The NAD^+^/NADH ratio is regarded as a measure of global brain energy capacity because the activities of many rate-limiting enzymes involved in the tricarboxylic acid cycle, ketone production, and glycolysis are regulated by this ratio [6–8].

Detection of changes in the NAD+/NADH ratio as well as other critical phosphorous-containing energy metabolites has been quantified using ^31^Phosphorous magnetic resonance spectroscopy (^31^P-MRS) imaging in living organisms, including humans. ^31^P-MRS imaging has consistently demonstrated brain energetic impairments in individuals with PD or with MS [3, 9–14]. Importantly, restoration of NAD^+^ levels was associated with a correction of energetic and mitochondrial deficits in PD-patient derived induced pluripotent stem cell (iPSC) dopaminergic neurons, and was associated with improved dopaminergic (DA) neuron survival and improved motor behavior in a PD animal model [15]. In multiple sclerosis (MS), we have demonstrated that treatment of two independent animal models of demyelination with orally delivered CNM-Au8, which raises intracellular NAD^+^ levels in target oligodendrocyte cells [16], markedly enhanced remyelination in both the corpus callosum and spinal cord lesions [17]. This remyelination was demonstrated to favorably impact function, as recovery of behavioral phenotypes in the cuprizone mouse model were observed with CNM-Au8 treatment [17]. These results, along with similar results from other independent studies, indicate that NAD metabolism is an important therapeutic target for both MS and PD [9, 18, 19].

CNM-Au8 is an orally-administered, concentrated suspension of clean-surfaced catalytically-active gold nanocrystals, whose surfaces catalyze the rapid oxidation of NADH to NAD^+^, increasing the intracellular availability of both NAD^+^ and ATP in in vitro studies [16, 17]. In multiple preclinical studies, CNM-Au8 has not only been shown to restore energetic homeostasis, but also reduce oxidative stress via superoxide dismutase-like and catalase-like activity. CNM-Au8 is blood-brain barrier penetrant and cell membrane-permeant. While nanoparticles of gold have been shown to have the capacity to act as biological catalysts [20, 21], to our knowledge, CNM-Au8 is the only suspension of gold nanocrystals with both exceptionally high catalytic activity and very low toxicity that is being developed as a disease-modifying agent for neurodegenerative disease. Preclinical efficacy studies in multiple in vitro and in vivo models have demonstrated CNM-Au8 enables remyelination of demyelinated axons and enhances the survival of a variety of neuronal subtypes in several neurodegenerative disease models [16, 17].

^31^Phosphorous magnetic resonance spectroscopy (^31^P-MRS) allows for the non-invasive assessment of phosphorous-containing energy metabolites, including ATP, of the whole brain in vivo. Ultrahigh-field scanners at 7 Tesla provide a substantial signal-to-noise gain, shortened (T1) relaxation time, and improved spectral resolution of phosphorous metabolites [3, 22] compared to lower strength scanners. Using ^31^P-MRS, brain levels of key energy metabolites such as ATP, total nicotinamide adenine dinucleotide [NAD(H)], phosphocreatine (PCr), inorganic phosphate (Pi), phospholipid monoester metabolites phosphocholine (PC) and phosphoethanolamine (PE), phospholipid diester metabolites glycerophosphocholine (GPC) and glycerophosphoethanolamine (GPE), and the nucleotide sugar, uridine diphosphate glucose (UDPG), can be measured. Separation of the NAD(H) signals into the reduced (NADH) and oxidized (NAD^+^) forms is technically challenging, but with 7T scanners, spectral editing techniques allow measurement of each metabolite as well as the NAD^+^/NADH ratio [23, 24].

The objective of the REPAIR clinical trial program was to demonstrate the effects of CNM-Au8 on brain energy metabolites (i.e., CNS target engagement) and evaluate safety in two sister studies that enrolled participants with either multiple sclerosis (REPAIR-MS, NCT03993171), or Parkinson’s disease (REPAIR-PD, NCT03815916). Brain energy metabolites were quantified using 7T ^31^P-MRS at baseline, before the start of treatment, and after 12 or more weeks of daily oral dosing with 15 mg or 30 mg CNM-Au8. End of treatment results at week 12 were compared to pre-treatment baseline in 24 participants with evaluable post-baseline scans, consisting of 13 participants from REPAIR-PD, and 11 participants from REPAIR-MS. The REPAIR-MS protocol incorporated a 6-week drug withdrawal period that followed the 12-week dosing period. Nine participants in the REPAIR-MS trial had a final ^31^P-MRS scan performed after completing the 6-week drug withdrawal period, allowing a comparison of brain energy metabolite levels after 12 weeks of continuous dosing to metabolite levels following drug withdrawal.

## Methods

### Standard protocol approvals, registrations, and patient consents

Study protocols were reviewed and approved by the Institutional Review Board of the University of Texas Southwestern Medical Center and were conducted in accordance with Good Clinical Practice Guidelines of the International Conference on Harmonization and the ethical principles of the Helsinki Declaration of 1975, as revised in 2000 [25]. REPAIR-PD was registered on clinicaltrials.gov with registration number NCT03815916 and REPAIR-MS with registration number NCT03993171 prior to the enrollment of the first participants for each trial. All participants provided written consent prior to participating in the study.

### Participant selection

Detailed inclusion/exclusion criteria are given in Tables S1 (REPAIR-PD) and S2 (REPAIR-MS). Key entry criteria for each REPAIR cohort are provided below.

REPAIR-PD: Participants were men or women aged 30–80 years old with a diagnosis of PD according to the MDS Clinical Diagnostic Criteria for Parkinson’s disease [26]. Additionally, participants were Hoehn and Yahr stage ≤ 3, had PD disease duration of ≤ 3 years, and were currently undergoing treatment with dopaminergic medications for at least 12 weeks with no change in the dose for at least 6 weeks prior to enrollment. Exclusion criteria included Montreal Cognitive Assessment (MoCA) < 18, unstable medical conditions, HIV, hepatitis B or C, clinically significant abnormalities on labs or electrocardiogram (ECG), current participation in other investigational drug studies, positive urine screen for drugs of abuse or alcohol abuse, individuals unable or unwilling to use acceptable forms of birth control during and for 6 months after completion of participation, positive pregnancy test, history of allergy to gold in any form, or deemed to be at risk for suicide.

REPAIR-MS: Participants were men or women aged 18–55 years old with a diagnosis of relapsing MS according to the revised McDonald Criteria [27], within 15 years of diagnosis at the time of screening. Additionally, participants were receiving stable treatment with natalizumab, defined as a stable dose maintained at the standard infusion interval of 28-days (± 5 days) for at least the prior six months, and were exhibiting stable disease activity based on the investigators’ judgment over the prior three months. Exclusion criteria included a clinical relapse requiring systemic steroid treatment within the prior three months, current treatment with any MS therapy other than natalizumab, any active ophthalmological cause for retinal damage other than MS, any ophthalmic disease or severe refractive defects that might confound the study results or optical coherence tomography assessment, unstable medical conditions, HIV, hepatitis B or C, clinically significant abnormalities on labs or ECG, current participation in other investigational drug studies, positive urine screen for drugs of abuse or alcohol abuse, individuals unable or unwilling to use acceptable forms of birth control during and for 6 months after completion of participation, positive pregnancy test, history of allergy to gold in any form, or deemed to be at risk for suicide.

### Trial design

REPAIR-PD and REPAIR-MS were developed as joint proof-of-concept studies to assess the CNS metabolic effects and safety of CNM-Au8 in participants diagnosed with either Parkinson’s disease or relapsing MS. Both studies were carried out by investigators at the University of Texas Southwestern Medical Center (UT Southwestern) who were, along with participants and all staff at the study site, blinded to CNM-Au8 dose level.

Participants with early, stable PD (i.e., not requiring dose adjustments to any dopaminergic medication during the study) were enrolled in REPAIR-PD. Participants with a diagnosis of stable relapsing MS within 15 years of screening were enrolled in REPAIR-MS. Participants were required to maintain the same the dose of any background therapy (e.g., natalizumab, symptomatic dopaminergic drugs) throughout the studies. All participants received CNM-Au8 in a volume of 120 mL, dispensed in two single-dose 60 mL food-grade high density polyethylene containers, with the instructions to drink both 60 mL bottles each morning on an empty stomach.

Participants were followed over 12 consecutive weeks during which study visits were conducted at 2–4 week intervals. A phone assessment was conducted at Week 2 to determine safety and tolerability. At Weeks 4, 8, and 12, clinical visits were conducted for pharmacokinetics (PK), pharmacodynamics (PD) biofluid sampling, and safety assessments with additional clinical assessments to evaluate secondary outcomes, described below.

^31^P-MRS scans were performed prior to initiation of treatment at the baseline visit and at end of study, which was scheduled to occur at week 12. A third scan was performed on participants in the REPAIR-MS trial after 6 weeks of drug withdrawal (from Week 12 to Week 18).

Beginning with the baseline visit, participants had ECG, safety labs, concomitant medication assessments, physical exams, treatment emergent adverse event assessments, scoring of Movement Disorder Society-Sponsored Revision of the Unified Parkinson’s Disease Rating Scale (MDS-UPDRS) for participants with PD and scoring of Expanded Disability Rating Scale (EDSS) for participants with MS, patient global impression of change and severity (PGI-C, PGI-S), clinician global impression of change and severity (CGI-C, CGI-S), and the Columbia suicide severity rating scale (C-SSRS) assessments performed at each visit until completion of their participation. For the time-and-event schedules for REPAIR-PD and REPAIR-MS, please see Supplemental Tables S3 and S4, respectively. MedDRA Version 22.0 was used for treatment emergent adverse event reporting.

### Interventions

Participants took the assigned dose of CNM-Au8 once daily for at least 12 weeks. Study investigators and participants were blinded to the dose of CNM-Au8 selected by the sponsor, which could range from 7.5 mg to 60 mg CNM-Au8; the blind will be maintained until another companion cohort of the REPAIR-MS trial in non-active progressive multiple sclerosis, which is currently underway, is completed.

### Protocol deviation due to Covid-19

Due to administrative suspension of in-person research visits at UT Southwestern in the Spring of 2020 during the COVID-19 pandemic, several patients continued treatment with the study drug for longer than the originally planned 12 weeks. Some study visit data that required an in-person visit was missing due to the COVID-19 related research suspension, but all participants had baseline and end-of-study primary and secondary outcome imaging assessments performed per protocol once in-person imaging was allowed. The end-of-study imaging assessment was performed 12–16 weeks post-baseline for all but two participants, one in REPAIR-PD (end-of-study imaging performed at 25 weeks) and one in REPAIR-MS (end-of-study imaging performed at 20 weeks).

### Imaging protocol and data processing

^31^P-MRS scans were performed at the baseline and Week 12 visits. Because some Week 12 ^31^P-MRS scans were delayed due to COVID-19 related research restrictions, they were considered as the Week 12 end of study imaging scan, irrespective of the actual timing.

For all scans, participants were positioned head-first and supine in a human 7T MRI scanner (Achieva, Philips Healthcare, Best, The Netherlands), operated at 120.6 MHz for ^31^P resonance, and located at the Advanced Imaging Research Center, UT Southwestern. The back of the head was positioned in the center of the detection radiofrequency (RF) coils (see below for details). Cushioned pads provided comfort and helped to secure the positioning of the head to reduce potential movement throughout the collection of data. Participants were reminded to remain awake during the collection of the ^31^P-MRS scan and with patient consent, body weights were added as needed to reduce body movement.

The 3D ^31^P MRSI data were acquired from the whole brain using a ^31^P transmit-receive (T/R) bird-cage volume RF coil of diameter 23 cm and length 10 cm (Gorter Center, Leiden University Medical Center, The Netherlands). The ^31^P coil was inserted into a single-channel bird-cage quadrature ^1^H T/R head coil (Nova Medical, Wilmington, MA, USA) for ^1^H-based B0 shimming (by the second-order pencil-beam projection method). After an initial scout image scan, multi-slice MPRAGE MRI images were collected for planning the shimming box and the MRSI data acquisition matrix. The shimming quality was checked by a non-localized ^31^P spectrum pulse acquired with the following parameters: TR 1.0 s, number of sampling points (NP) 4 k, effective excitation bandwidth 3.2 kHz, spectral bandwidth (BW) 8 kHz, number of acquisitions (NA) = 80. The 3D ^31^P MRSI data were acquired using a block pulse, at TR 0.5 s, flip angle 55^o^, in-plane resolution 2 × 2 cm^2^, reconstructed to 1 × 1 cm^2^, slice thickness 2 cm, k-space acquisition weighting (α = 1.7 and β = 1.0), elliptic k-space sampling, 4k sampling points zero-filled to 8k prior to Fourier transformation, typical coronal imaging FOV (FH × RL × AP) = 10 × 16 × 16 cm^3^ (with phase encoding steps 5 × 8 × 8), and NA = 24. A non-localized whole-brain scan was also performed with a pulse-acquire sequence at TR = 0.52 s and NA = 160 for checking spectral quality.

A half-cylinder-shaped ^1^H/^31^P dual-tuned T/R partial volume RF coil (Philips Healthcare, Best, The Netherlands) was used to image the posterior brain (occipital and parietal lobes) for evaluation of the brain redox state based on the measurement of NAD^+^/NADH ratio. Axial and sagittal T2-weighted spin-echo multi-slice MRI images were collected from the posterior head for planning volume-based shimming. Typical ^31^P-MRS data acquisition parameters were utilized including TR 1.0 s, a hard readout pulse at B_1_ 59 µT with minimum TD of 0.17 ms, NP 4k and zero filled to 8k, and BW 8 kHz. Data were accumulated in NA blocks of 256 or 512 and later summed, upon aligning at the reference PCr ^31^P signal, to yield a reference spectrum. Despite the dispersing power of ultrahigh field 7T, the conventional pulse-acquire sequence fails to deliver a brain ^31^P spectrum with a clearly resolved NAD signal due to overlapping from the α-ATP signal, which is several-fold larger, broad-based, and typically asymmetrical in lineshape. To obtain ^31^P MRS spectra with resolved NAD from overlapping α-ATP signals by spectral editing, an additional ^31^P MRS spectrum was acquired using an inversion-recovery (IR) sequence with the same TR and TD but contained an adiabatic inversion pre-pulse followed by a short delay of 0.37 to selectively nullify the NAD signal. The resultant standalone α-ATP signal with well-defined spectral baseline in the IR spectrum was then utilized to remove the overlapping α-ATP signal from the reference spectra for obtaining the resolved NAD signal. Given that UDPG and its structural analogues also contribute a set of ^31^P signals in the upfield of α-ATP, their spectral effect on the NAD signal was also corrected prior to the deconvolution of NAD into NAD^+^ and NADH as described previously [24, 28]. For quantitative comparison of different metabolites in the posterior brain region, an additional scan was performed, using the partial volume coil, under the fully relaxed condition with a long TR of 15 s at a flip angle of 55^o^.

The time-domain ^31^P FID data were post-processed (zero-filling, apodization, Fourier transformation, and zero- and first-order phasing) using the scanner software (SpectroView, Philips Healthcare). The first three sampling points were discarded to remove the broad background phospholipid signal. The frequency-domain ^31^P spectra were analyzed by quantification of metabolite ^31^P signals using an in-house program written in MATLAB (MathWorks, Natick, MA, USA). Peak area for each metabolite was normalized to the PCr peak area. Prior-knowledge about chemical shift and J-coupling constant of NAD^+^ and NADH were used as initial values in the lineshape fitting [24]. Normalized ^31^P signal intensity ratios were obtained for each metabolite in reference to PCr for both REPAIR cohorts at each time point, and the entire data analysis was blinded to the operators. Mean levels of each metabolite at the end of study (Week 12) visit were compared to the respective baseline value for each subject. Comparative analyses were performed separately for data collected using the partial volume coil and data collected using the whole brain coil.

### ^*31*^*P-MRS imaging outcome measures*

The primary outcome was mean change from baseline to end-of-study visit for the NAD^+^:NADH ratio. Secondary endpoints included the mean change from baseline to the end of study visit in the fraction (%) of NAD^+^ of total NAD peak area, and in the fraction (%) of NADH of total NAD peak area. Mean change in NAD^+^/NADH ratio from Week 12 to Week 18 is also reported for the MS cohort.

Several bioenergetic metabolite exploratory outcomes, functional exploratory outcomes, and safety outcomes were also evaluated. Here, we report the regression of baseline values versus mean percentage change of the average brain β-ATP signal, which is regarded to reflect brain ATP levels due to the lack of overlap with overlapping phosphorous peaks [29]. Only the β-ATP signal arises from pure ATP, whereas the α-ATP peak includes small contributions from NAD^+^/NADH and α-ADP, and γ-ATP contains a contribution from β-ADP. Analyses of other phosphorous metabolites will be reported in a separate publication when data from the ongoing REPAIR-MS (Cohort 2) trial can be evaluated coordinately with Cohort 1. REPAIR-MS (Cohort 2, NCT03993171) is currently enrolling participants with non-active secondary progressive MS or primary progressive MS as a companion study to REPAIR-MS (Cohort 1) for relapsing MS. Similarly, functional exploratory outcomes specific to MS, including change from baseline to end-of-study in the EDSS score and measures of low contrast letter acuity, fine and gross motor control, and cognition for MS, will be reported in a separate publication along with results from Cohort 1. Exploratory clinical outcomes for PD included change from baseline to end-of-study in the validated clinical rating scale MDS-UPDRS, as well as measures of gait, balance, and motility for PD.

Safety was assessed via spontaneously reported adverse events, serious adverse events, discontinuations due to adverse events, deaths, and the Columbia Suicide Severity Rating Scale (C-SSRS).

### Statistical analysis

The sample size for this proof-of-concept study was calculated based on the observed variance in brain metabolite levels in a prior ^31^P-MRS study involving a cohort of 7 healthy volunteers in which repeat ^31^P-MRS scans were taken two weeks apart [24]. The ratio of the NAD^+^/NADH baseline average value was 4.09 ± 0.78, while the repeat ^31^P-MRS assessment was 4.11 ± 0.79. The standard deviation of the difference from baseline to week 2 was 0.28 and the coefficient of variation was 5.8% ± 4.8%, demonstrating consistent ^31^P-MRS NAD^+^/NADH reproducibility. We originally hypothesized a mean difference from baseline to end-of-study of 0.41 (10%) based on an average baseline NAD^+^:NADH redox value of 4.1, and an estimated standard deviation for the difference from baseline of 0.42. Assuming a paired t-test, power of 80%, and a two-sided alpha of 0.05, this resulted in a sample size estimate of 11 participants, which was increased by two to a planned enrollment of 13 participants per cohort to account for possible dropouts. While the REPAIR studies were being conducted, advances in ^31^P-MRS imaging techniques by Ren et al. improved the specificity of the NAD^+^:NADH measure [24] but also resulted in increased intra-participant variance, thereby increasing the required target sample size given the same assumed effect size. Due to COVID-19 related challenges, sample sizes for the respective studies were not revised from the original plan. Instead, the statistical analysis plan was designed to include a pre-specified integrated analysis of both disease cohorts for the primary outcome assessment.

Paired t-tests were used to assess for change in NAD^+^:NADH ratio, NAD^+^ fraction, and NADH faction, comparing baseline to end-of-study. Paired t-test was used to assess change in NAD^+^/NADH ratio from Week 12 to Week 16 in the MS cohort. Linear regression was used to assess participants’ percent change from baseline to end-of-study ATP levels compared their baseline ATP levels, as well as for participants’ percent change from baseline to end-of-study brain phosphorylation potential compared their baseline phosphorylation potential.

Paired t-tests were also calculated for each individual gait or balance parameter reported by the APDM Mobility Lab Timed Up and Go (TUG), Walk, and Sway tests which were performed at baseline and on the end-of-study visit. Sidak’s correction for the APDM variables was employed to account for the large number of comparisons resulting in a significance threshold of 0.001. MDS-UPDRS parts 1, 2, and 4 were analyzed with ANOVA, while part 3 and total score was assessed using a mixed-effects model to account for the missing data caused by missed in-person visits related to the COVID-19 research suspension. Statistical analyses were performed using SAS 9.4 (SAS Institute, Inc., Cary, NC). Figures were generated in Prism 9.4.1 (Graphpad Software LLC, San Diego, CA).

## Results

### Participants

Demographics, participant disposition and baseline clinical features of the 13 participants in REPAIR-PD are shown in Table 1. Participants were enrolled in the study from October 2019 through November 2020, and the last participant completed the study in April 2021. All participants completed the baseline and end-of-study visit, and the overall adherence of participants based on consumed bottle counts was 97%.

**Table 1:** Baseline characteristics of participants in REPAIR-PD (n = 13)

Baseline Participant Demographics	
Age (years)	65.9 ± 7.8
Sex (% Male)	54%
Race/ethnicity, non-Hispanic White (%)	92%
Age at diagnosis (years)	64.5 ± 8.1
Time from diagnosis at enrollment (months)	17.2 ± 8.8
MMSE	28.8 ± 1.3
MDS UPDRS Part I	4.8 ± 3.4
MDS UPDRS Part II	3.0 ± 2.6
MDS UPDRS Part III	20.5 ± 8.1
MDS UPDRS Part IV	1.0 ± 1.5
MDS UPDRS Total	29.4 ± 9.3
Average Levodopa Dose (in IR equivalents, mg)	532 ± 267
# of patients on Carbidopa/Levodopa only	9 (70%)
# of patients on MAO-B inhibitor (rasagiline)	3 (23%)
# of patients on dopamine agonist (ropinirole)	1 (7.7%)

Values given are mean ± standard deviation. Levodopa dose is calculated as (mg immediate-release levodopa) + (mg controlled-release levodopa tablets)0.75 + (mg extended-release capsules)0.7.

Demographics, participant disposition and baseline clinical features of the 11 participants in REPAIR-MS are shown in Table 2. Participants were enrolled in the study from December 2019 through February 2021, and the last participant completed the study in July 2021. All participants completed the baseline and end-of-study visit, and the overall adherence of participants based on consumed bottle counts was 86%.

**Table 2:** Baseline characteristics of participants in REPAIR-MS (n = 11)

Baseline Participant Demographics	
Age (years)	41.6 ± 10.0
BMI (kg/m^2^)	27.9 ± 5.7
Sex (% Female)	69%
Race/ethnicity, non-Hispanic White (%)	85%
Age at diagnosis (years)	34.5 ± 9.4
Time from symptom onset at enrollment (years)	7.1 ± 4.6
Time since last relapse (years)	4.5 ± 3.2
% Treated with natalizumab	100%
EDSS Score	2.7 ± 1.9

Values given are mean ± standard deviation.

### Brain energy metabolites

**Fig. 1:**
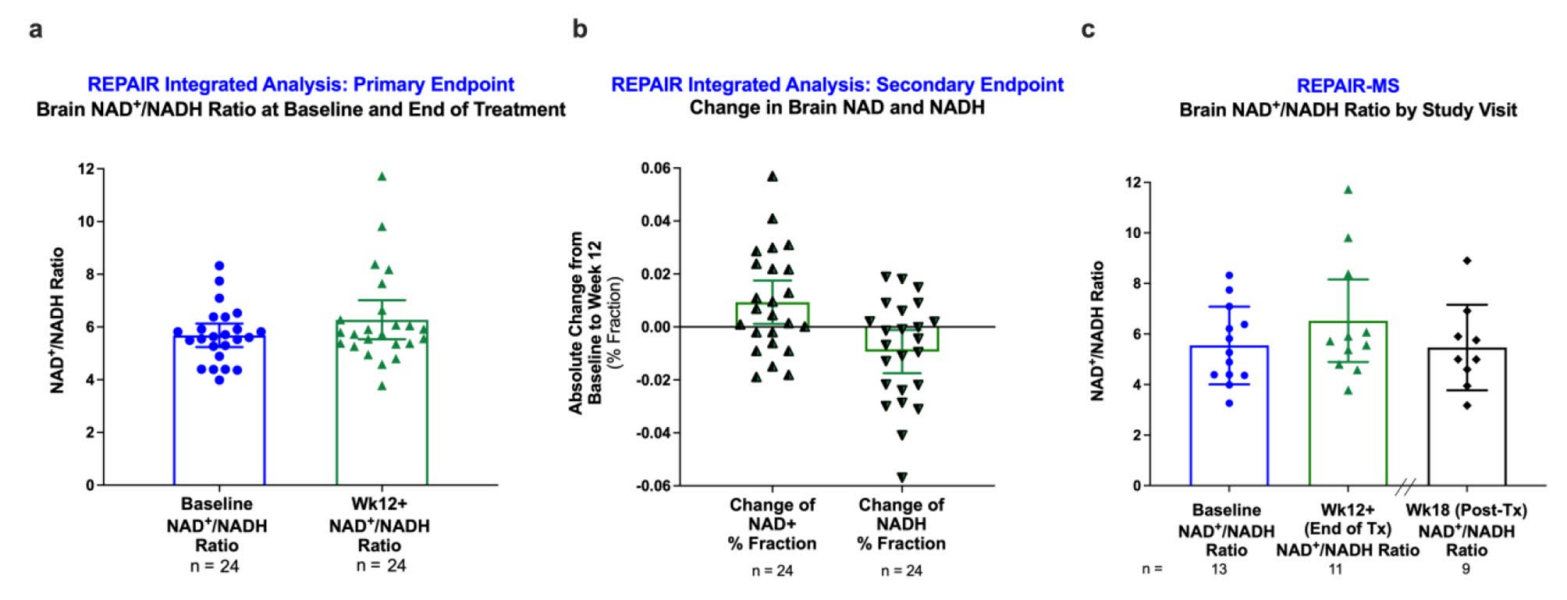
Increased NAD^+^/NADH Brain Ratio in Both PD and MS Participants in the REPAIR Studies. **(a-c)** Pre-specified integrated analyses of REPAIR-MS and REPAIR-PD values of the primary endpoint, **(a)** change in brain NAD^+^/NADH ratio [mean change in ratio from baseline to end of study = 0.5891, + 10.4%, *p* = 0.0371 (paired t-test)], and secondary endpoints, **(b)** change in NAD^+^ fraction and change in NADH fraction after 12 + weeks of daily dosing of CNM-Au8 [mean change of NAD + fraction, 0.0093, + 1.1%, with a reciprocal change of NADH fraction, -0.0093, -1.1%; *p* = 0.0264 (paired t-test)]. **(c)** The REPAIR-MS trial protocol included a 6-week withdrawal of treatment following 12 weeks of dosing. After withdrawal of treatment, at week 18, a final ^31^P-MRS scan showed that NAD^+^/NADH ratio had returned to baseline levels. Bar graphs show mean ±95% CI; individual participant values at baseline (blue circles), end of study (green triangles), and 6 weeks post-treatment (black diamonds)

The results for the prespecified primary endpoint, the mean change in the brain NAD^+^/NADH ratio across the two cohorts demonstrated a statistically significant increase by an average of 0.589 (+ 10.4%) following 12 weeks of treatment with CNM-Au8 (*p* = 0.037, paired t-test) (Fig. 1a). Key secondary endpoints, mean change from baseline in the NAD^+^ and NADH fractions of the total NAD pool, were concordant with the primary endpoint, in that the NAD^+^ fraction increased (*p* = 0.026) while the NADH fraction decreased (*p* = 0.026) (Fig. 1b). The results for each study also independently demonstrated consistencies supporting an increase in the NAD^+^/NADH ratio, with results of 0.386 (+ 6.8%, *p* = 0.108, paired t-test); and 0.83 (+ 14.3%, *p* = 0.145, paired t-test) for REPAIR-PD and REPAIR-MS, respectively, although neither cohort on its own demonstrated statistical significance.

The REPAIR-MS trial protocol included a 6-week withdrawal of treatment following 12 weeks of dosing. After withdrawal of CNM-Au8 treatment from week 12 to week 18, the final ^31^P-MRS scan showed that NAD^+^/NADH ratio had returned to baseline at Week 18 (Fig. 1c).

**Fig. 2:**
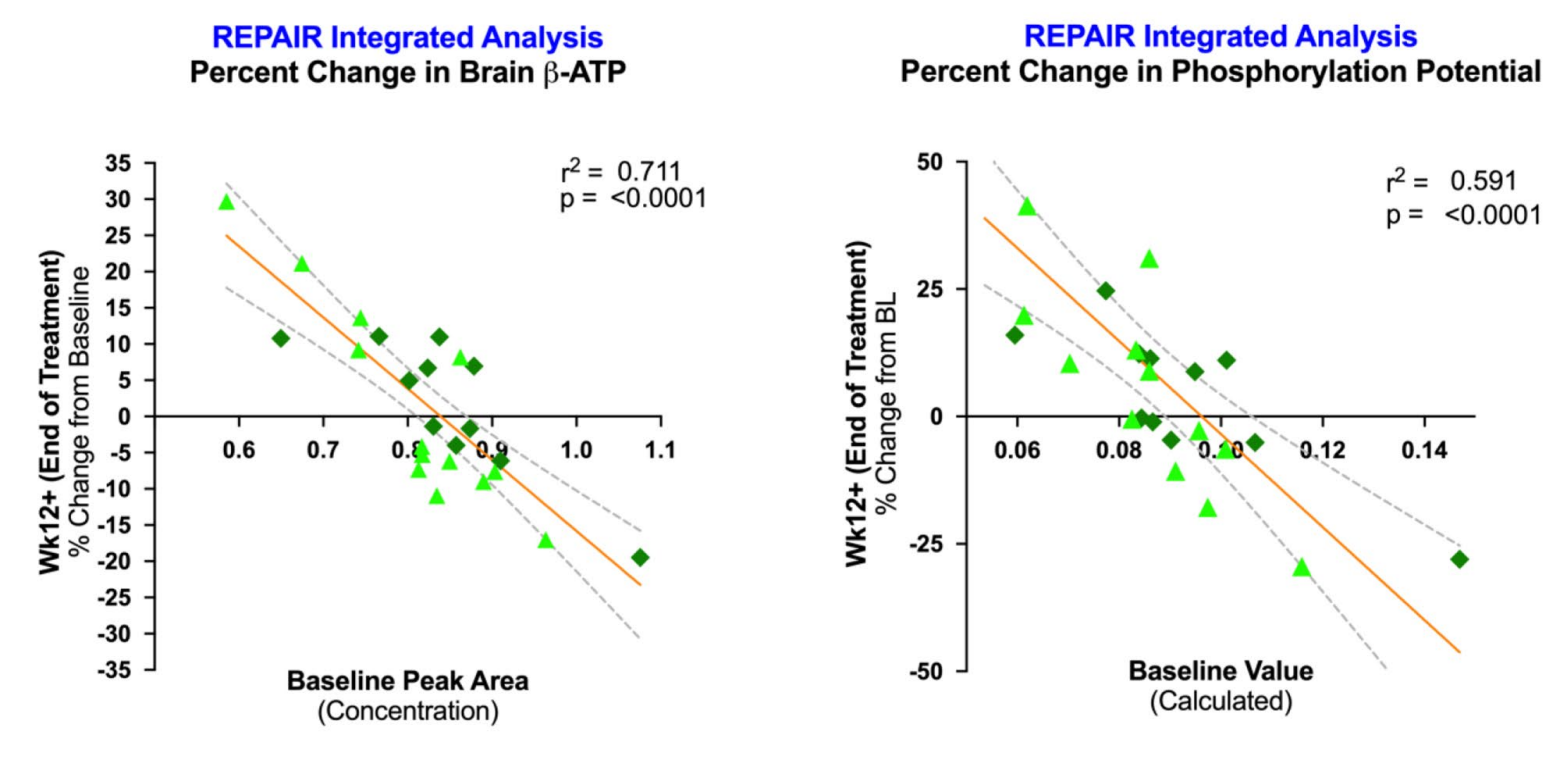
**ATP and Phosphorylation Potential Equilibration in REPAIR Clinical Trials.** Pre-specified integrated analyses of REPAIR-MS and REPAIR-PD values of the exploratory endpoints, (a) Percent change in β-ATP from baseline to end-of-study and (b) Percent change in phosphorylation potential from baseline to end-of study, plotted against baseline values for β-ATP and phosphorylation potential, respectively. Individual values for REPAIR-PD participants (n = 13) are shown as light green triangles; individual values for REPAIR-MS participants are shown as dark green diamonds. Linear regression line (orange) and 95% CI (dashed curves) are shown

Analyses of prespecified exploratory endpoints demonstrated that homeostatic equilibrium was achieved for both adenosine triphosphate (ATP), and the phosphorylation potential index [β-ATP/(ADP*Pi(in))], a ratio of high energy to low energy phosphates [30] (Fig. 2). For this metabolite energy index, the percent change from baseline to week 12 was significantly inversely correlated with baseline levels, such that participants with relatively lower baseline levels demonstrated increases, and participants with relatively higher baseline levels demonstrated a re-balancing effect with levels equilibrating to the baseline population mean. This relationship was observed in both REPAIR-PD and REPAIR-MS, and on an integrated basis across the two studies for: β-ATP (r^2^ = 0.711, *p* < 0.0001; Fig. 2a), and phosphorylation potential (r^2^ = 0.591, *p* < 0.0001; Fig. 2b).

### Exploratory endpoints

MDS-UPDRS is a clinician-administered, validated instrument for measurement of both non-motor and motor aspects associated with PD for use in research and clinical trials [31]. The MDS-UPDRS is comprised of four parts: (i) non-motor experiences of daily living; (ii) motor experiences of daily living; (iii) motor examination; (iv) motor complications. Analysis of variance of the MDS-UPDRS parts revealed a statistically significant improvement in the part 2 score (Supplemental Fig. 1A) driven mainly by a significant improvement at week 4 (mean part 2 score at baseline = 3.0, week 4 = 1.8, end-of-study = 3.2, mean difference = -1.2, ANOVA with Dunnett’s correction, *p* = 0.03). No significant change was noted in the MDS-UPDRS total score (Supplemental Fig. 1B) nor in any other part. Dosage of dopaminergic drugs was held constant in all subjects during the study.

The APDM Mobility lab generated 32 unique parameters for the Sway test, 7 for the TUG test, and 51 for the Walk test. After Sidak correction, none of these demonstrated a statistically significant mean change from baseline to end of study.

### Safety

A total of 8 (61.5%) REPAIR-MS participants and 6 (46%) REPAIR-PD participants experienced at least one treatment emergent adverse event (TEAE). All TEAEs were reported as mild or moderate; no participants experienced TEAEs reported as severe. No participants experienced TEAEs that were considered related to study drug by the study investigators. There were no serious adverse events (SAEs), no TEAEs resulting in study discontinuation, and no deaths. TEAEs occurring in more than one participant across both disease cohorts included sinusitis (n = 3), nasopharyngitis (n = 3), and paresthesia (n = 2). Other safety parameters that were assessed included hematology, serum chemistry, urinalysis, physical exam, electrocardiogram, and the CSSRS. CSSRS data revealed no suicidal ideation from any participants across both studies. Of the laboratory assessments, only one clinically significant finding (elevated creatine kinase) was reported in the REPAIR-MS cohort for a participant at the baseline visit. The safety lab was collected prior to participant dosing with CNM-Au8 and was found to resolve within two weeks at this participant’s next visit, while treated with CNM-Au8. The participant’s creatine kinase remained within the normal range throughout the remainder of the study. In conclusion, CNM-Au8 was well-tolerated, and no safety signals were observed in either cohort.

## Discussion

The objective of our study was to demonstrate brain target engagement of orally administered CNM-Au8 in participants with PD or MS using 7T ^31^P-MRS. After 12 + weeks of daily oral dosing of CNM-Au8, a 10.4% increase in brain NAD^+^/NADH ratio compared to baseline was observed in a prespecified integrated analysis of the two disease cohorts. This statistically significant observation was supported by an end-of-study increase in NAD^+^ levels and a concomitant decrease in NADH levels compared to baseline. When each PD or MS cohort was analyzed separately, an increase in NAD^+^/NADH ratio was consistently observed in each cohort, although these increases did not independently reach statistical significance, likely due to the limited sample size. The REPAIR-MS trial protocol included a 6-week withdrawal of treatment following 12 weeks of dosing. After 6-week withdrawal of CNM-Au8 treatment from week 12 to week 18, a final ^31^P-MRS scan showed that NAD^+^/NADH ratio had returned to baseline pre-treatment levels. This result indicated that CNM-Au8 dosed daily for at least 12-weeks increased brain NAD^+^/NADH ratio, an effect that ceased following withdrawal of CNM-Au8.

Limitations of our study include the relatively small number of participants in the REPAIR trials. In addition, the studies had to be terminated after evaluating only one dose of CNM-Au8 due to enrollment challenges associated with the COVID-19 pandemic. While future studies with larger cohorts of participants using brain metabolite imaging to evaluate CNM-Au8 treatment effects are warranted, there were several important observations that these studies afforded, despite their small size.

A previous ^31^P-MRS study demonstrated that the brain NAD^+^/NADH ratio decreases approximately 0.5% per decade in healthy individuals [23]. Based on this observation, the result of a 10.4% increase in NAD^+^/NADH ratio in 12–16 weeks with CNM-Au8 treatment represents a comparatively large treatment effect, one that may be sufficient to have clinical impact on energy metabolic-related declines in neurodegenerative disease. To our knowledge, CNM-Au8 is the only drug in development with catalytic activity targeted at intracellular NADH conversion to NAD^+^ with a demonstrated, potentially beneficial, effect on these metabolites in participants with neurodegenerative disease.

The REPAIR studies also extend the findings of the recent NADPARK study, which reported that elevating brain total NAD levels by dietary supplementation with a high-dose NAD precursor in 30 participants with PD for 30 days was associated with mild clinical improvement, increased brain glucose metabolism, and decreased inflammatory cytokines in serum and CSF [32]. The study was limited by use of a 3T ^31^P-MRS scanner that precluded measurement of the NAD^+^/NADH redox ratio in the study’s participants. Because our study employed a 7T scanner we were able to demonstrate that CNM-Au8 treatment affected NAD at the granularity of increasing the NAD^+^/NADH ratio, which has been identified as holding ameliorative potential for correcting metabolic defects due to impaired mitochondrial electron transport chain activity beyond what is possible with NAD precursor supplementation [33, 34].

The REPAIR studies also demonstrated significant effects of CNM-Au8 on ATP levels and on the phosphorylation potential using the full volume coil. Global assessment of brain metabolite changes may provide a measure of the overall burden of disease on the brain, independent of specific brain regions, and thus be more comparable across studies and disease states [35]. We observed a significant relationship between the percent change in brain ATP level (baseline to end-of-study) and baseline ATP levels. Participants with low ATP at baseline exhibited increased ATP at the end of the study, while those with high ATP at baseline had reduced ATP at study end. A similar relationship was observed between percent change in brain phosphorylation potential and participants’ baseline phosphorylation potential. Phosphorylation potential, an index of bioenergetic state reflecting the amount of free energy readily available to cells, has been demonstrated to be lower in patients with PD [30], mitochondrial myopathy [36], and Leber’s hereditary optic neuropathy [37], all diseases with mitochondrial impairment.

We believe these observations demonstrate an important homeostatic energetic effect of CNM-Au8 in these patient populations. Both increased and decreased levels of brain ATP are indicative of disease state, in both PD and MS. In PD, a low ATP/Pi ratio is seen in Parkinson’s disease [38] and a concurrent FDG-PET study showed this change accompanies reduction in rate of glucose uptake [39]. Thus, in PD patients with low ATP or phosphorylation potential, the increase in ATP levels by end of study could be considered therapeutic. Likewise, in MS patients, brain ATP levels correlated with MS disease severity as measured by EDSS suggesting brain energy metabolic support may be an important therapeutic target [13]. Further, PD patients have been shown to have resting brain ATP levels that are lower in PD brain compared to matched healthy controls, while the ATP forward rate constant is higher in PD brains compared to controls, indicating the existence of a cellular energy compensatory mechanism whereby brain cells attempt to maintain ATP homeostasis by increasing ATPase activity and ATP production [3]. High ATP levels were also reported in PD patients homozygous for PINK1 mutations which are known to cause dysfunction of the mitochondrial respiratory chain [40]. The elevated ATP concentration seen in these participants was hypothesized to result from astrocytic glycolysis in the setting of neuronal degeneration [40]. Astrocytes, serving as supportive cells, provide trophic metabolites and ATP to diseased neurons, and in response to exigent neuronal energetic needs, can transition to a glycolytic and oxidative energy-producing “overdrive” state to pump out excess ATP [41]. In this theory of attempted ATP compensation, the finding of high concentrations of ATP in a PD brain could point to neuronal crisis, rather than health [40]. Therefore, our finding of CNM-Au8-related homeostatic effects on β-ATP, as well as on the related phosphorylation potential, is noteworthy and warrants further investigation.

This short-duration study was underpowered to detect clinical benefit with CNM-Au8 treatment, and thus the finding of no significant clinical benefit (as manifested by stable MDS-UPDRS total scores and APDM gait/balance parameters) was anticipated. We attribute the statistically significant improvement in MDS-UPDRS part 2 (driven by improvement at 4 weeks) to a placebo effect induced by expectation of benefit by subjects who knew they were taking active drug. Historically, symptomatic effects, i.e., effects with short-lived beneficial impact that converge to the level of placebo with time and that lack evidence of longer-term neuroprotection, have confounded outcome measure interpretation for putative neuroprotective drugs [42, 43]. In this context, we view the lack of such a symptomatic effect of clinical improvement of CNM-Au8 during this short trial as an advantage for the continued development of this putative disease modifying agent.

## Conclusions

CNM-Au8 is a nanomedicine being developed to treat failures in energy metabolism associated with many neurodegenerative diseases. Its novel mechanism of action includes targeting the redox couple NAD^+^ and NADH, the key drivers of ATP production and utilization in all cells. Three double-blind, randomized, placebo-controlled Phase 2 clinical trials have been conducted investigating the safety and efficacy of CNM-Au8 to treat amyotrophic lateral sclerosis and multiple sclerosis, with long-term extension studies associated with these trials currently ongoing. Results from the REPAIR studies indicate that, in concordance with preclinical studies, brain energy metabolism is favorably modulated in individuals with MS or PD. Together with the emerging results from the placebo-controlled trials [44], CNM-Au8 represents a strong candidate drug with appropriate target engagement and safety data supporting its advancement Phase 3 trials for the treatment of neurodegenerative disease.

### Electronic supplementary material

Below is the link to the electronic supplementary material.

Supplementary Material 1

## Data Availability

All datasets used and/or analysed during this study are either included in this published article and its supplementary information files, or are available as de-identified, HIPAA-compliant datasets upon reasonable request to the corresponding author. To protect the privacy of individuals who participated in this study, a fully executed data transfer agreement between Clene Nanomedicine, Inc., and the third party may be required. A control dataset used for power calculation and refinement of the ^31^P-MRS analysis of NAD + and NADH levels was published as a pilot study preceding this work. These data are available from: Ren J, Malloy CR, Sherry AD. Quantitative measurement of redox state in human brain by ^31^P MRS at 7T with spectral simplification and inclusion of multiple nucleotide sugar components in data analysis. Magn Reson Med 2020;84:2338–51. 10.1002/mrm.28306. [Ref [24]].

## List of abbreviations

^31^P-MRS: ^31^Phosphorous-Magnetic resonance spectroscopy
ATP: Adenosine triphosphate
BW: Bandwidth
C-SSRS: Columbia Suicide Severity Rating Scale
CGI-C: Clinician Global Impression of Change
CGI-S: Clinician Global Impression of Severity
CNS: Central nervous system
DA: Dopaminergic
ECG: Electrocardiogram
EDSS: Expanded Disability Rating Scale
GPC: Glycerophosphocholine
GPE: Glycerophosphoethanolamine
HIV: Human immunodeficiency virus
iPSC: Induced pluripotent stem cell
IR: Inversion-recovery
MDS-UPDRS: Movement Disorder Society-Sponsored revision of the Unified Parkinson’s Disease Rating Scale
MoCA: Montreal Cognitive Assessment
MS: Multiple sclerosis
NA: Number of acquisitions
NAD+: Oxidized form of nicotinamide adenine dinucleotide
NADH: Reduced form of nicotinamide adenine dinucleotide
NP: Number of sampling points
PC: Phosphocholine
PCr: Phosphocreatine
PD: Parkinson’s Disease
PD: Pharmacodynamics
PE: Phosphoethanolamine
PGI-C: Patient Global Impression of Change
PGI-S: Patient Global Impression of Severity
Pi: Inorganic phosphate
PK: Pharmacokinetics
RF: Radiofrequency
SAE: Serious adverse event
SD: Standard deviation
T/R: Transmit/receive
TEAE: Treatment emergent adverse event
TUG: Timed Up and Go
UDPG: Uridine diphosphate glucose
