# Correction: Evidence of brain target engagement in Parkinson’s disease and multiple sclerosis by the investigational nanomedicine, CNM- Au8, in the REPAIR phase 2 clinical trials

**DOI:** 10.1186/s12951-023-02269-4

**Published:** 2024-01-03

**Authors:** Jimin Ren, Richard B. Dewey, Austin Rynders, Jacob Evan, Jeremy Evan, Shelia Ligozio, Karen S. Ho, Peter V. Sguigna, Robert Glanzman, Michael T. Hotchkin, Richard B. Dewey, Benjamin M. Greenberg

**Affiliations:** 1 Department of Neurology, University of Texas Southwestern Medical Center, 5323 Harry Hines Blvd., Dallas, TX 75390 USA; 2 Present Address: Parkinson’s Disease and Movement Disorders Center, Boca Raton, FL 33486 USA; 3 3Clene Nanomedicine, Inc., 6550 S Millrock Dr., Suite G50, Salt Lake City, UT 84121 USA; 4 Instat Clinical Research, A Veristat Company, 1 Wilson St., Chatham, NJ 07928 USA

**Correction: Journal of Nanobiotechnology (2023) 21:473**

10.1186/s12951-023-02236-z

Following publication of the original article [1], the authors reported that the 2nd author was omitted from the author group. Richard B. Dewey III has been added to the author group both in the original article and in this correction article.

In addition, the authors identified an error in affiliation 1. The affiliation was incorrectly given as “Department of Neurology, University of Texas Southwestern Medical Center, 5323 Harry Hines Blvd, Dallas, TX 75390, USA”, but should have been “University of Texas Southwestern Medical Center, Department of Neurology, 5323 Harry Hines Blvd., Dallas, TX 75390”. This error is corrected in the affiliations list below and in the original article.
