# Supplementary material for: Evidence of brain target engagement in Parkinson’s disease and multiple sclerosis by the investigational nanomedicine, CNM-Au8, in the REPAIR phase 2 clinical trials
Source: J Nanobiotechnology. 2023 Dec 13;21:478. doi: 10.1186/s12951-023-02236-z (PMC10717868; doi:10.1186/s12951-023-02236-z)
Supplement: Supplementary file 1 — Supplementary Material 1 [file 12951_2023_2236_MOESM1_ESM.docx]

**Evidence of Brain Target Engagement in Parkinson’s Disease and Multiple Sclerosis by the Investigational Nanomedicine, CNM-Au8, in the REPAIR Phase 2 Clinical Trials**

Jimin Ren^1^

Richard B. Dewey III^1,2^

Austin Rynders^3^

Jacob Evan^3^

Jeremy Evan^3^

Shelia Ligozio^4^

Karen S. Ho^3,*^

Peter V. Sguigna^1^

Robert Glanzman^3^

Michael T. Hotchkin^3^

Richard B. Dewey, Jr.^1,2^

Benjamin M. Greenberg^1^

^1^ University of Texas Southwestern Medical Center, Department of Neurology, 5323 Harry

Hines Blvd., Dallas, TX 75390

^2^ Present address: Parkinson’s Disease and Movement Disorders Center, Boca Raton, FL, 33486

^3^ Clene Nanomedicine, Inc., 6550 S Millrock Dr., Suite G50, Salt Lake City, UT 84121

^4^Instat Clinical Research, A Veristat Company, 1 Wilson St., Chatham, NJ 07928

^*^Corresponding author, karen@clene.com, 6550 Millrock Dr., Suite G50, Salt Lake City, UT 84121.

**SUPPLEMENTARY MATERIALS**

**Table S1. Inclusion/Exclusion criteria for REPAIR-PD**

| **Inclusion Criteria** | The patients to be enrolled in this study must meet the following inclusion criteria:   1. Able to understand and give written informed consent and follow study procedures. 2. Male or female, aged 30 – 80 years or age (inclusive) at the time of PD diagnosis. 3. PD subjects will be recruited in accordance with the MDS Clinical Diagnostic Criteria for PD:    1. Parkinsonism present (bradykinesia + either rest tremor or rigidity)    2. 2 of the following 4 supportive criteria:       1. Clear and dramatic beneficial response to dopaminergic medication       2. Presence of levodopa induced dyskinesias       3. Rest tremor of a limb       4. Olfactory loss or cardiac sympathetic denervation seen on prior MIBG SPECT 4. Duration of PD since diagnosis is < 3 years (inclusive) 5. Modified Hoehn and Yahr stage < 3 6. Treatment with dopaminergic therapy for at least 12-weeks and with no change in current medications within the prior 6-weeks |
| --- | --- |
| **Exclusion Criteria** | Patients will be excluded from the study if they meet any of the following criteria:   1. Atypical parkinsonism, including that due to drugs, metabolic disorders, encephalitis, cerebrovascular disease, normal pressure hydrocephalus, or other neurodegenerative disease. 2. The presence of any of the following:   Unequivocal cerebellar abnormalities  Downward vertical gaze limitation or slowing of downward saccades  Diagnosis of behavioral variant frontotemporal dementia or primary progressive aphasia  Parkinsonian features restricted to the lower limbs for > 3 years  Treatment with dopamine blockers or depleters in a time course consistent with drug induced parkinsonism  Absence of an observable response to high dose levodopa despite moderate disease severity  Expert considers a diagnosis of alternative syndrome more likely than PD  Rapid progression of gait impairment requiring wheelchair within 5 years of onset  Complete absence of progression of motor symptoms over 3 years unless due to treatment  Early bulbar dysfunction within the first 5 years since diagnosis  Inspiratory respiratory dysfunction (stridor or frequent sighs)  Severe autonomic failure in the first 5 years  Recurrent falls (>1 per year) because of impaired balance in the first 3 years  Disproportionate dystonic anterocollis or hand contractures of hands or feet within 10 years  Absence of any of the common non-motor features of PD despite 5 years of disease  Otherwise unexplained pyramidal tract signs (weakness, hyperreflexia, or extensor toe signs)  Bilateral symmetric parkinsonism   1. Mini-Mental State Examination (MMSE) score of less than 19. 2. Patient with a history of any clinically significant or unstable medical condition based on the Investigator’s judgment. 3. History of human immunodeficiency virus (HIV), hepatitis C (HepC) virus antibody, or hepatitis B (HepB) virus antibody. 4. Based on the investigator’s judgment, patients who may have difficulty complying with the protocol and/or study procedures. 5. Patient with clinically significant abnormalities in hematology, blood chemistry, ECG, or physical examination not resolved by the Baseline visit which according to Investigator may interfere with study participation. 6. Patients with clinically significant hepatic or renal dysfunction or clinical laboratory findings that would limit the interpretability of change in liver or kidney function, or those with low platelet counts (<150 x 10^9^ per liter) or eosinophilia (absolute eosinophil count of ≥500 eosinophils per microliter) at Screening. 7. Patient participating in any other investigational drug trial or using investigational drug (within 12 weeks prior to screening and thereafter) 8. Positive screen for drugs of abuse or known history of alcohol abuse. 9. Women of child-bearing potential, or men, who are unwilling or unable to use accepted methods of birth control during the study and for 6 months following completion of study participation. 10. Women with a positive pregnancy test, are lactating, or are planning to become pregnant during the study or within 6 months of the end of this trial. 11. Patients with implanted metal objects in their body that may be affected by an MRI procedure. 12. Patients who are claustrophobic or otherwise unlikely to be able to complete the MRI scanning procedures. 13. History of allergy to gold in any form. 14. Patient is considered a suicide risk in the opinion of the Investigator, has previously made a suicide attempt, or is currently demonstrating active suicidal ideation. Subjects with intermittent passive suicidal ideation are not necessarily excluded based on the assessment of the Investigator. |

**Table S2. Inclusion/Exclusion criteria for REPAIR-MS**

| **Inclusion Criteria** | Patients enrolled in *Cohort 1* (relapsing MS) must meet the following inclusion criteria:   1. At least 18 years of age and up to 55 years (inclusive) of age at Screening. 2. Clinical diagnosis of Relapsing Multiple Sclerosis (RMS) (meeting McDonald criteria, 2017). 3. Diagnosis of MS no longer than 15 years prior to Screening. 4. Stable treatment with natalizumab, defined as a stable dose maintained at the standard infusion interval of 28-days (±5 days) for at least the prior six (6) months. 5. Stable disease activity based on the Investigator’s judgment over the prior three (3) months. 6. Any hematological parameters and/or biochemical parameters that fall outside the Within Normal Limits range at Screening must be assessed as Not Clinically Significant (NCS) and deemed stable or transient in nature. 7. Able to understand and give written informed consent.   Patients enrolled in *Cohort 2* (non-active progressive MS) must meet the following inclusion criteria:   1. Male or female, aged 18 – 70 years or age (inclusive); 2. Diagnosis of primary progressive multiple sclerosis (PPMS) or non-active secondary progressive multiple sclerosis (SPMS), according to revised 2017 McDonald criteria at the Screening visit; 3. EDSS score at the Screening visit of less than or equal to 6.5 (inclusive); 4. Participants must be taking either B-cell depleting therapy (e.g., ocrelizumab, rituximab) or S1P modulator therapy (e.g., siponimod) with consistent stable dosing for at least 48 weeks prior to the Screening visit; 5. Any hematological parameters and/or biochemical parameters that fall outside the Within Normal Limits range at Screening must be assessed as Not Clinically Significant (NCS) and deemed stable or transient in nature; and 6. Able to understand and give written informed consent. |
| --- | --- |
| **Exclusion Criteria** | Patients in *Cohort 1* (relapsing MS) will be excluded from the study if they meet any of the following criteria:   1. Patients with a clinical relapse requiring systemic steroid treatment within the prior three (3) months. 2. Patients treated with any other MS therapy other than natalizumab; or treated with clemastine fumarate. 3. Based on the Investigator’s judgment, patients with a history of significant other major medical condition that may interfere with the conduct of the study or interpretation of the study results. 4. Based on the Investigator’s judgment, patients who may have difficulty complying with the protocol and/or study procedures. 5. History of any clinically significant abnormality in hematology, blood chemistry, ECG, or physical examination not resolved by the Baseline visit which according to Investigator can interfere with study participation. 6. Patients with clinically significant hepatic or renal dysfunction or clinical laboratory findings that would limit the interpretability of change in liver or kidney function, or those with low platelet counts (< 150 x 109 per liter) or eosinophilia (absolute eosinophil count of ≥500 eosinophils per microliter) at Screening. 7. Patients with a prior history of, or positive serological assay for the presence of HIV infection, or laboratory evidence of active or chronic infection with hepatitis C (HCV) or hepatitis B (HBV). Note, participants who have been vaccinated for HBV and have detectable HB antibodies are not excluded unless positive for hepatitis surface antigen (HBsAg). 8. Patients participating in any other investigational drug trial or using an investigational drug (within 12 weeks prior to screening and thereafter). 9. Positive screen for drugs of abuse or known alcohol abuse. 10. Females who are pregnant, have a positive pregnancy test, are nursing, or who plan to get pregnant during the course of this clinical trial or within 6 months of the end of this trial. 11. Women of child-bearing potential, or men, who are unwilling or unable to use accepted methods of birth control during the study and for 6 months following completion of study participation. 12. Patients with implanted metal objects in their body that may be affected by an MRI procedure. 13. Patients who are claustrophobic or otherwise unlikely to be able to complete the MRI scanning procedures. 14. Patients with a history of gold allergy. 15. Patient is considered a suicide risk in the opinion of the Investigator, has previously made a suicide attempt, or is currently demonstrating active suicidal ideation. Subjects with intermittent passive suicidal ideation are not necessarily excluded based on the assessment of the Investigator. 16. Any active ophthalmological cause for retinal damage other than MS (e.g. cataracts, uveitis, macular degeneration, macular exudate, macular edema, glaucoma, severe astigmatism, ocular trauma, neuromyelitis optica, ischemic optic neuropathy, congenital nystagmus, retinal detachment, amblyopia, optic disk drusen). 17. Severe refractive defects: refractive errors (–5 dioptres to +5 dioptres or more in either eye, or axial eye length >26 mm), hypermetropia (> 5 dioptres; cylinder > 3 dioptres); or based on the Investigator’s judgment any other ophthalmic diseases that might confound the study results or optical coherence tomography assessment. 18. PRN use of stimulant medications including: amphetamine, dextroamphetamine, lisdexamfetamine, methylphenidate, or modafinil; however, stimulant medications, taken on a consistent daily dose for at least 12-weeks are allowed. No changes in the dose of any stimulant medications are allowed during the study |

**Supplementary Materials**

**Table S3. Time and Event Schedule for REPAIR-PD**

| **Time and Events Schedule** | **Visit** | **-1** | **0** | **1** | **2** | **3** | **4** | **5** |
| --- | --- | --- | --- | --- | --- | --- | --- | --- |
|  | **Phase** | **Screening** | **Baseline** | **Treatment Period** | | | | **EOS** |
|  | **Week** | **-6** | **0** | **2** | **4** | **8** | **12** | **16** |
|  | **Day** | -42 to -1 | 1^a^ | 14 ^a^ | 28 ^a^ | 56 ^a^ | 84 ^a^ | 112 ^b^ |
| ICF Signed | | X |  |  |  |  |  |  |
| Eligibility Review | | X | X |  |  |  |  |  |
| Medical History | | X |  |  |  |  |  |  |
| ConMed/Prior Med Assessment | | X | X | X | X | X | X | X |
| Physical Examination | | X | X |  | X | X | X | X |
| Height Assessment | | X |  |  |  |  |  |  |
| Weight Assessment | | X | X |  |  |  | X |  |
| Urine Drug Test | | X |  |  |  |  |  |  |
| HIV/Viral Hepatitis Screen | | X |  |  |  |  |  |  |
| Serum Pregnancy Test ^c^ | | X |  |  |  |  |  |  |
| Urine Pregnancy Test ^c^ | |  | X |  | X | X | X |  |
| Vital Signs | | X | X |  | X | X | X | X |
| 12‑lead ECG ^d^ | | X | X |  | X | X | X | X |
| Clinical Laboratory (Blood) | | X | X |  | X | X | X | X |
| Urinalysis | | X | X |  | X | X | X | X |
| MMSE | | X |  |  |  |  |  |  |
| Treatment Assignment | |  | X |  |  |  |  |  |
| Dispense/Return Drug | |  | X |  | X | X | X |  |
| PK Sampling (Whole Blood) | |  |  |  | X^e^ | X^e^ | X^f^ | X^e^ |
| PD Sampling (Whole Blood, Plasma) | |  | X^e^ |  | X^e^ | X^e^ | X^e^ |  |
| CSF Sampling (optional) | |  | X |  |  |  | X |  |
| Adverse Events | | X | X | X | X | X | X | X |
| Anxiolytic Administration | |  | X |  |  |  | X |  |
| ^31^P-MRS | |  | X |  |  |  | X |  |
| MDS-UPDRS | |  | X |  | X | X | X |  |
| PGI | |  | X |  | X | X | X |  |
| CGI | |  | X |  | X | X | X |  |
| C-SSRS | | X | X | X | X | X | X | X |
| APDM Instrumented Tests | |  | X |  |  |  | X |  |
| Phone call | |  |  | X |  |  |  |  |

**Time and Events Schedule Footnotes:**

1. Scheduled Visit ± 4 days for Visit 1-4. For Visit 0 (Baseline), the ^31^P-MRS and PD CSF assessments may be completed over a -7 day window prior to Day 1. All assessments must be completed prior to administration of first study drug dose.
2. Timing for the EOS assessment should occur at four weeks (±3 days) from last dose regardless of early termination or completion of the trial.
3. For females of child bearing potential only.
4. Electrocardiogram (ECG) intervals will be summarized and presented descriptively. ECG rhythm will be interpreted by the Investigator as normal (N), abnormal not-clinically significant (aNCS), or abnormal clinically significant (aCS). Triplicate values will be collected at Baseline and averaged for comparison to single assessments at subsequent visits.
5. Whole blood for PK and PD will be taken pre-dose only (~1 hour prior to the dose of study drug).
6. Whole blood for PK will be taken at pre-dose (T_0_) and at 1, 2, 4, and 6 hours after dosing for the visit. The exact time at which the patient took his/her previous day’s study drug dose must be recorded in order to impute a 24-hour trough value (T_24-imputed_).

**Table S4. Time and Event Schedule for REPAIR-MS**

| **Time and Events Schedule** | **Visit** | **-1** | **0** | **1** | **2/3** | **4** | **5/6** | **7** |
| --- | --- | --- | --- | --- | --- | --- | --- | --- |
|  | **Phase** | **Screening** | **Baseline** | **Treatment Period** | | | **EOS** | |
|  | **Week** | **-6** | **0** | **2** | **4/8** | **12** | **14/16** | **18** |
|  | **Day** | -42 to -1 | 1^a^ | 14 ^a^ | 28/56 ^a^ | 84 ^a^ | 98/112 ^b^ | 126 ^b^ |
| ICF Signed | | X |  |  |  |  |  |  |
| Eligibility Review | | X | X |  |  |  |  |  |
| Medical History | | X |  |  |  |  |  |  |
| ConMed/Prior Med Assessment | | X | X | X | X | X |  | X |
| Physical Examination | | X | X |  | X | X |  | X |
| Height Assessment | | X |  |  |  |  |  |  |
| Weight Assessment | | X | X |  |  | X |  |  |
| Urine Drug Test | | X |  |  |  |  |  |  |
| HIV/Viral Hepatitis Screen | | X |  |  |  |  |  |  |
| Serum Pregnancy Test ^c^ | | X |  |  |  |  |  |  |
| Urine Pregnancy Test ^c^ | |  | X |  | X | X |  |  |
| Vital Signs | | X | X |  | X | X |  | X |
| 12‑lead ECG ^d^ | | X | X |  | X | X |  | X |
| Clinical Laboratory (Blood) | | X | X |  | X | X |  | X |
| Urinalysis | | X | X |  | X | X |  | X |
| Treatment Assignment | |  | X |  |  |  |  |  |
| Dispense/Return Drug | |  | X |  | X | X |  |  |
| PK Sampling (Whole Blood) | |  |  |  | X^e^ | X^e^ | X | X |
| PD Sampling (Whole Blood, Plasma, Urine) | |  | X^e^ |  | X^e^ | X^e^ |  | X |
| CSF Sampling (optional) | |  | X ^a^ |  |  | X |  |  |
| Adverse Events | | X | X | X | X | X | X | X |
| ^31^P-MRS (7T) | |  | X ^a^ |  |  | X |  | X |
| MRI (3T) | |  | X ^a^ |  |  | X |  |  |
| OCT | |  | X |  |  | X |  | X |
| Visual Acuity (LCLA/HCVA) | |  | X |  | X | X |  | X |
| 9-Hole Peg Test | |  | X |  | X | X |  | X |
| Timed 25-Foot Walk Test | |  | X |  | X | X |  | X |
| SDMT | |  | X |  | X | X |  | X |
| EDSS | |  | X |  | X | X |  | X |
| PGI | |  | X |  | X | X |  | X |
| CGI | |  | X |  | X | X |  | X |
| C-SSRS | | X | X |  | X | X |  | X |
| Phone call | |  |  | X |  |  |  |  |
|  | |  |  |  |  |  |  |  |

**Time and Events Schedule Footnotes:**

1. Scheduled Visit ± 4 days. For Visit 0 (Baseline), the ^31^P-MRS, MRI, and/or PD CSF assessments may be completed over a (-7) day window prior to Day 1. All Baseline assessments must be completed prior to administration of first IP dose.
2. Timing for the Weeks 14 and 16 PK collection and conduct of the Week 18 EOS visit should occur at two, four, and six weeks (±4 days), respectively, from last dose for patients completing the 12-week treatment period. For patients prematurely discontinuing the study, the EOS visit will be conducted at 4 weeks (±4 days) from their last dose of study medication and will not include PK, PD, Visual Acuity, Global Impressions, OCT, 9-Hole Peg Test, Timed 25-Foot Walk Test, SDMT, EDSS or ^31^P MRS imaging. Further, the Weeks 14 and 16 PK collection visits will not occur of those who do not complete the treatment phase.
3. For females of child bearing potential only.
4. Electrocardiogram (ECG) intervals will be summarized and presented descriptively. ECG rhythm will be interpreted by the Investigator as normal (N), abnormal not-clinically significant (aNCS), or abnormal clinically significant (aCS). Triplicate values will be collected at Baseline and averaged for comparison to single assessments at subsequent visits.
5. Whole blood for PK and PD (including urine) will be taken pre-dose only (~1 hour prior to the dose of IP).

**Supplemental Figure 1. MDS-UPDRS Part 2 score and B. Total score at each visit.**

**
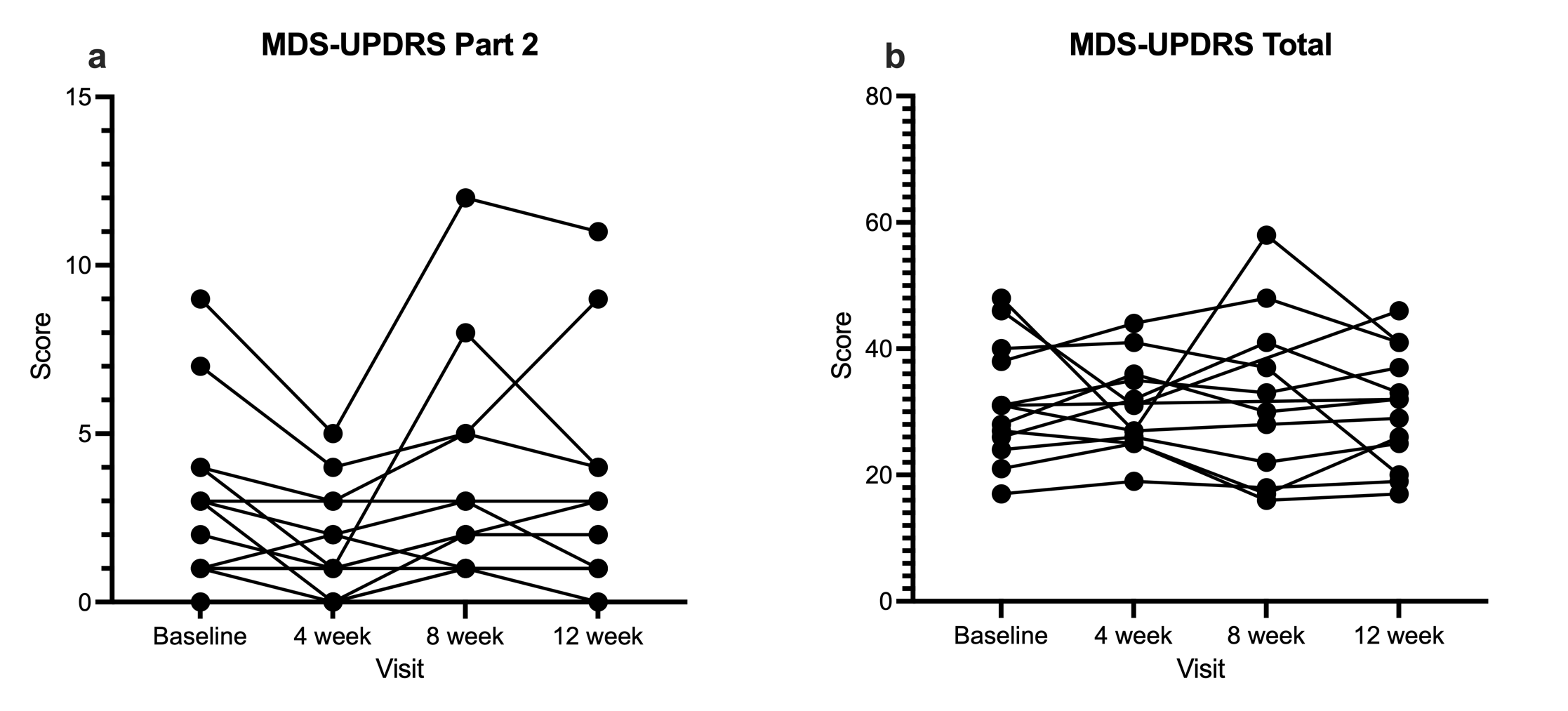
**
